# Targeting Cattle-Borne Zoonoses and Cattle Pathogens Using a Novel Trypanosomatid-Based Delivery System

**DOI:** 10.1371/journal.ppat.1002340

**Published:** 2011-10-27

**Authors:** G. Adam Mott, Raymond Wilson, Anuruddika Fernando, Ailie Robinson, Paula MacGregor, David Kennedy, Dick Schaap, Jacqueline B. Matthews, Keith R. Matthews

**Affiliations:** 1 Centre for Immunity, Infection and Evolution, Institute for Immunology and Infection Research, School of Biological Sciences, University of Edinburgh, Edinburgh, United Kingdom; 2 Moredun Research Institute, Pentlands Science Park, Edinburgh, United Kingdom; 3 Intervet Schering-Plough, Boxmeer, The Netherlands; Yale University, United States of America

## Abstract

Trypanosomatid parasites are notorious for the human diseases they cause throughout Africa and South America. However, non-pathogenic trypanosomatids are also found worldwide, infecting a wide range of hosts. One example is *Trypanosoma (Megatrypanum) theileri*, a ubiquitous protozoan commensal of bovids, which is distributed globally. Exploiting knowledge of pathogenic trypanosomatids, we have developed *Trypanosoma theileri* as a novel vehicle to deliver vaccine antigens and other proteins to cattle. Conditions for the growth and transfection of *T. theileri* have been optimised and expressed heterologous proteins targeted for secretion or specific localisation at the cell interior or surface using trafficking signals from *Trypanosoma brucei*. In cattle, the engineered vehicle could establish in the context of a pre-existing natural *T. theileri* population, was maintained long-term and generated specific immune responses to an expressed *Babesia* antigen at protective levels. Building on several decades of basic research into trypanosomatid pathogens, *Trypanosoma theileri* offers significant potential to target multiple infections, including major cattle-borne zoonoses such as *Escherichia coli*, *Salmonella* spp., *Brucella abortus* and *Mycobacterium* spp. It also has the potential to deliver therapeutics to cattle, including the lytic factor that protects humans from cattle trypanosomiasis. This could alleviate poverty by protecting indigenous African cattle from African trypanosomiasis.

## Author Summary

Single-celled parasites of the order Kinetoplastida are responsible for devastating diseases of humans and animals, including African trypanosomiasis, Chagas' disease and leishmaniasis. However, there are also many species of trypanosomatids that do not cause disease and are distributed globally. One example is *Trypanosoma (Megatrypanum) theileri*, which is restricted to bovids and ubiquitous in cattle herds worldwide. This organism is maintained extracellularly in the blood and tissues long-term without any observed ill effects on host health or productivity. Using knowledge of gene expression and protein trafficking in pathogenic trypanosomatids, we have successfully developed, from first principles, *Trypanosoma theileri* as a delivery system for vaccine antigens and therapeutics. Procedures for the growth, transfection and heterologous gene expression of *T. theileri* have been developed, and the delivery of a vaccine antigen derived from *Babesia divergens* evaluated *in vivo*. Our results demonstrate the ability of *T. theileri* to be used as a flexible and easily manipulated protein delivery system suitable for the control of cattle pathogens and cattle-borne zoonoses. In one notable application, we propose that the system could allow the expression of serum trypanolytic factors in cattle, with the potential to alleviate poverty in Africa through the killing of pathogenic trypanosomatids in livestock.

## Introduction

Human health is intimately linked to animal health through the impact of infectious agents on livestock productivity and their potential for zoonosis [1]. Indeed, animal borne disease represents the major source of both emergent and resurgent pathogens in humans, this affecting communities in both the developed and developing world. One major source of such zoonotic infections is cattle, which threaten human health in the developed world through their capacity to transmit bacterial infections including *Escherichia coli*, *Salmonella* spp., *Campylobacter* spp., *Brucella* spp. and mycobacteria. In the developing world, livestock are also a reservoir for Human African Trypanosomiasis (HAT) caused by *Trypanosoma brucei rhodesiense.* This parasite, and *Trypanosoma brucei gambiense*, is closely related to *Trypanosoma brucei brucei*, which cannot infect humans. The basis of this host restriction is that human serum contains a trypanolytic component of high-density lipoprotein, ApoLI, that kills *T. brucei brucei* [2], but to which the human infective parasites have evolved resistance [3], [4].

Although trypanosomes are important causes of human and animal disease, many species are non-pathogenic. One of these is *Trypanosoma (Megatrypanum) theileri*, a cosmopolitan parasite of bovids that infects most cattle worldwide [5]–[11]. Although one report describes reduced milk yield in three infected cows [12], the ubiquity of infection with this organism in cattle herds suggests that it has no significant impact on health or productivity in healthy animals, while cases of disease in immuno-compromised animals are sufficiently rare as to merit individual case reports [13]–[15]. The parasite is transmitted in the contaminated faeces of tabanid flies and gains entry into the host through breaks in the skin or via contamination of the oral mucosa [16]. Thereafter, it lives extracellularly, being sustained at a low level (∼100 organisms/ml) for the life of the host. Being non-pathogenic, systemic, related to the genetically well-characterised *T. brucei*, and sustained long-term, we conceived that *T. theileri* would make a suitable protein delivery system in cattle, able to generate immunity to expressed antigens. As a naturally non-pathogenic kinetoplastid, *T. theileri* offers considerable advantages over alternative vaccine delivery systems that comprise engineered, attenuated pathogens such as *Salmonella* [17], [18], *Mycobacterium* [19], [20], *E. coli* [21], *Vibreo cholera*, *Listeria* or *Shigella* [22]. Furthermore, being maintained over long periods at low level, *T. theileri* offers the potential to generate sustained immune responses of greater efficacy than conventional vaccination approaches and to deliver therapeutic proteins of benefit to bovine health or to limit the zoonotic potential of cattle borne diseases.

## Results

**Figure 1:**
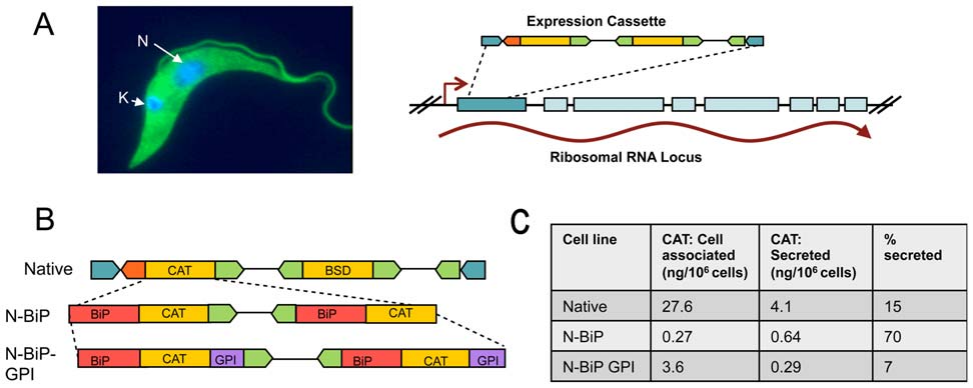
Heterologous proteins can be expressed and specifically localized in *T. theileri*. (**A**) An immunofluorescence image of *Trypanosoma theileri* stained with antibody to *T. brucei* alpha tubulin; the kinetoplast and nuclear DNA is stained with DAPI (blue). (**B**) Schematic representation of the integration of an expression cassette into the SSU rRNA locus of the *T. theileri* genome by homologous recombination. Read-through transcription drives expression from the integrated cassette. (**C**) Expression vectors used to assay CAT protein expression in *T. theileri*, this being as an unmodified protein (“Native”) or modified for secretion (“N-BiP”) or surface expression (“N-BiP-GPI”). For the N-BiP and N-BiP-GPI fusions, two gene copies were inserted in tandem to improve overall expression, these being separated by the *T. theileri* beta-alpha tubulin intergenic region. The respective distribution of CAT protein to the cell pellet or extracellular milieu is indicated on the right hand side, this being quantitated by CAT ELISA assay.

To develop *T. theileri* as a protein delivery system, the conditions for its axenic *in vitro* growth and genetic manipulation were established. To achieve this, a *T. theileri* isolate, originally identified as a contaminant of a primary bovine reticulocyte culture, was cultured under various conditions, optimal and sustained growth being achieved using a semi-defined medium containing 50% conditioned media from a bovine cell culture. In this medium, cell densities of 1×10^5^ – 2×106 cells/ml were achieved during routine passage (Figure S1 in Text S1). Under these conditions, the position of the kinetoplast (a specialised mitochondrial genome in trypanosomatid parasites; [23]) varied in relation to the cell nucleus but this was not clearly dependent upon the cell culture density (Figure 1A, Figure S1 in Text S1). To generate stable transfectants, bi-cistronic and tri-cistronic expression constructs were developed that comprised a drug selectable marker gene and a reporter gene, this being integrated into the small subunit 18S ribosomal RNA gene locus (Figure 1B). Here, RNA polymerase I-mediated read-through transcription can drive efficient gene expression, transcripts being processed and capped via trans splicing of a *T. theileri*-specific spliced leader (SL) RNA sequence, matching the situation in other kinetoplastids [24], [25]. This SL sequence was identified by 5′RACE of *T. theileri* transcripts and matched the determined SL sequence of one previously isolated *T. theileri* sample, *T. theileri* D30 (Figure S2 in Text S1; [26]), this matching the trypanosomatid consensus. In order to drive effective gene expression, RNA processing signals derived from *T. theileri* were used. Since almost no molecular information for these organisms was available, we isolated *T. theileri* intergenic sequences using degenerate primers able to amplify between the coding regions of well conserved genes (i.e. paraflagellar rod, tubulin, actin genes) predicted from the analysis of other kinetoplastid genomes to be tandemly arranged (Figure S3 in Text S1; [27]). The resulting expression constructs were transfected into *T. theileri* via nucleofector technology and selected using blasticidin or phleomycin drug selection. Unlike other kinetoplastid organisms, G418 and hygromycin B were not effective for selection in *T. theileri*, wild type cells exhibiting high levels of resistance to these drugs. This may have developed in natural *T. theileri* populations through long-term use of aminoglycoside antibiotics for the treatment of infections such as those responsible for bovine mastitis.

In order to develop the system for the optimal delivery of proteins to the bovine host, the potential to target expressed heterologous proteins to distinct cellular locations in *T. theileri* was investigated. For secretion, fusion proteins were created comprising the N-terminus of the *T. brucei* BiP/GRP78 protein (PMID:8227199) (N-BiP), which in *T. brucei* targets proteins for release into the extracellular milieu [28]. For cell surface expression, signals providing a C-terminal GPI-addition sequence were also added (N-BiP-GPI), whereas for internal expression, native proteins were expressed. Quantitative expression analysis of a chloramphenicol acetyl transferase (CAT) reporter protein confirmed protein expression and accurate targeting, with the percentage of protein released to the cell medium being 70% for the N-BiP fusion, 7% for the N-BiP-GPI fusion and 15% for the native protein, the latter probably including protein released from dead cells in the culture (Figure 1C). Although the overall expression efficiency was reduced considerably (10 to 50-fold) by the inclusion of heterologous targeting signals (N-BiP, N-BiP-GPI), this could be compensated somewhat by the expression of two copies of the reporter gene in tandem, generating a tri-cistronic construct (Figure 1C).

**Figure 2:**
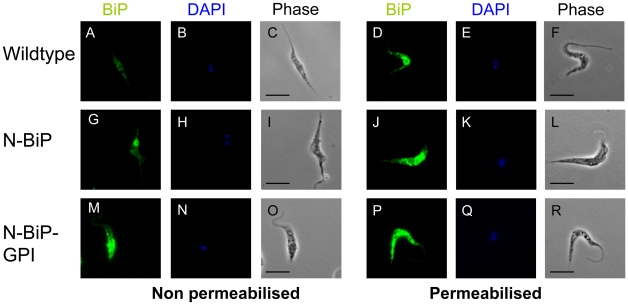
Cellular location of expressed N-BiP and N-BIP-GPI fusion proteins. Distribution of expressed Bd37 assayed via its fusion to *T. brucei* BiP and detected using a BiP-specific antibody. Cells were fixed with paraformaldehyde and then either 0.1% Triton-X100 permeabilised, or not, to distinguish intracellular from surface exposed protein following immunofluorescence. In wild-type cells, only weak staining was observed (**A–C**) in the absence of permeabilisation whereas permeabilisation (**D–F**) allows visualisation of endogenous, intracellular *T. theileri* BiP protein, which cross-reacts with the *T. brucei* BiP antibody. In contrast, the N-BiP-Bd37 fusion protein generates intense staining at the flagellar-pocket in intact cells (**G–I**), plus an intense intracellular staining in permeabilised cells comprised of endogenous BiP and N-BiP-Bd37 protein in the ER (**J–L**). Expression of the N-BiP-Bd37-GPI fusion protein generates a strong signal on intact cells indicating the presence of cell-surface protein (**M–O**); permeabilised cells also show strong straining representing the combined endogenous BiP and the expressed fusion protein **(P–R).** Camera exposures with respect to the BiP fluorescence and image handling were precisely consistent between cells under each condition to allow comparison of the relative intensity of signal. DAPI images reveal the kinetoplast and nuclear position, these often overlying one another. Scale Bar = 10 µm.

**Figure 3:**
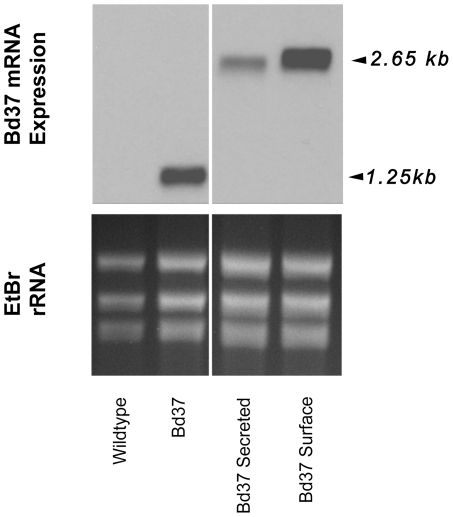
Transcript expression of exogenous genes in transgenic *T. theileri.* Northern blots of untransfected *T. theileri* and *T. theileri* engineered to express native Bd37, N-BiP Bd37 (‘Bd37 secreted’) or N-BiP Bd37 GPI (‘Bd37 surface’), transcripts being detected using a riboprobe detecting Bd37. Respective loading is indicated by the Ethidium bromide stained rRNA (lower panel).

To evaluate *T. theileri* as a vaccine delivery system, cells were engineered to express the Bd37 antigen from the cattle pathogen, *Babesia divergens* [29]. In general, protection against *Babesia* is thought to require components of the innate and adaptive immune systems (Reviewed in [30]). Effector arms of the adaptive immune system include antigen-specific antibodies, which have been hypothesized to target both infected erythrocytes and extracellular merozoites. There is also evidence that antibody-dependent cell-mediated cytotoxicity (ADCC) plays a role in parasite control during acute infection [31], [32]. A recombinant version of the Bd37 protein has been shown to stimulate antibody-directed protective immunity to *B. divergens* when administered in the context of an adjuvant [33], [34]. In our study, Bd37 protein was targeted for internal expression, surface localization or extracellular release by the expression of unmodified Bd37, N-BiP-Bd37-GPI or N-BiP-Bd37 fusion proteins. Since anti-Bd37 antibody was not effective in immunfluorescence assays, localisation was assessed for surface and secreted expression by incubating non-permeabilised or 0.1% TRITON X100 permeabilised cells with an antibody against *T. brucei* BiP, this recognising the N-terminal BiP component of the expressed fusion protein. In wild type cells, a signal was detected in permeabilised cells (Figure 2 D–F), reflecting the recognition of *T. theileri* BiP protein by antibody raised against *T. brucei* BiP. However, as expected, non-permeabilised cells showed little reactivity (Figure 2 A–C). In contrast, in transgenic *T. theileri* expressing Bd37, a strong punctate signal was detected in non-permeabilised cells for the N-BiP-Bd37-GPI fusion (Figure 2 M–O), whereas N-BiP-Bd37 cells exhibited a somewhat weaker signal concentrated close to the kinetoplast (Figure 2 G–I). This supported a surface-associated expression for the N-BiP-Bd37-GPI protein and flagellar pocket associated signal (the route for protein secretion in trypanosomatids) for N-BiP-Bd37, although staining of flagellar pocket-proximal vesicles cannot be excluded. Transcripts generated from the expressed reporter constructs were of the expected size, with the polyadenylation sites used for mRNAs derived from each of the constructs being approximately coincident with each other and with the site of beta tubulin polyadenylation (Figure 3 and Figure S4 in Text S1).

**Figure 4:**
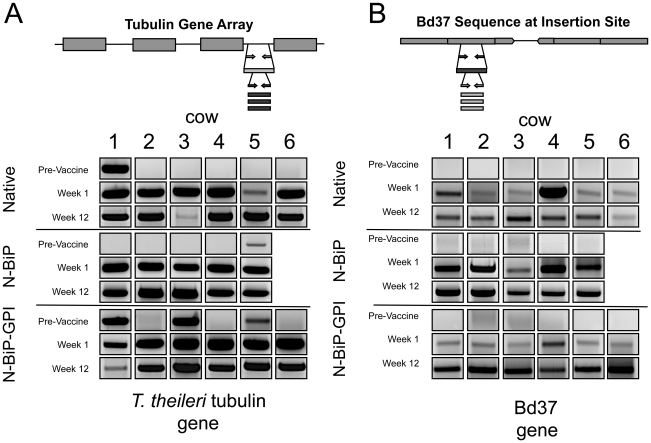
Inoculation with the vaccine vehicle results in successful establishment of the population in cattle. (**A**) Schematic representation of the amplicon used to detect *T. theileri* genomic DNA. The amplicon, specific for the *T. theileri* tubulin gene array, will detect both endogenous *T. theileri* infection and the presence of the transgenic vaccine vehicle. PCR products were derived from individual calves either before inoculation with transgenic *T. theileri*, or 1 week and 12 weeks post inoculation. Prior to inoculation 5/17 animals harboured a naturally occurring *T. theileri* population. (**B**) A second amplicon specific for the expressed Bd37 gene detects only genomic DNA from the transgenic *T. theileri.* The cattle all became positive for the transgenic vaccine vehicle within 1 week, regardless of their original infection status, this being sustained throughout the 12 weeks of the experiment for all animals.

**Figure 5:**
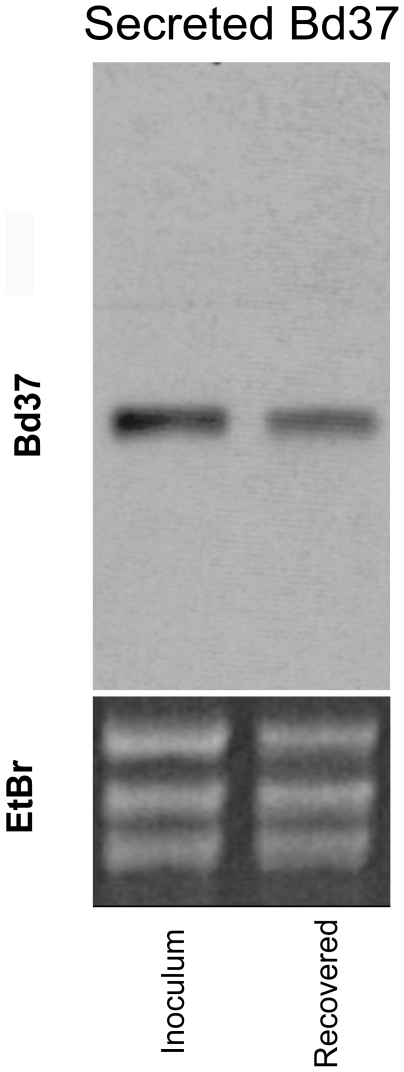
Sustained transgene expression in *T. theileri* after growth in cattle. Northern Blot to determine N-BiP Bd37 transcript expression in cultured *T. theileri* cells used to inoculate cattle, or harvested after 3 weeks growth in inoculated cattle. Expression of the Bd37 transcript was maintained. Respective loading is indicated by the Ethidium bromide staining rRNA (lower panel).

Having established protein localisation at different cellular locations, each *T. theileri* cell line was inoculated intravenously into groups of cattle (internal expression, n = 6 animals; secreted expression, n = 5 animals; surface expression, n = 6 animals), these being maintained in a fly-free, high containment facility. Of these, 5 of the 17 animals had been found to contain a pre-existing natural *T. theileri* population by screening blood using a specific, nested PCR assay for the *T. theileri* tubulin gene array (Figure 4A). Regardless of this, the transgenic *T. theileri* established in all animals within one week, these being detectable by nested PCR specific for the Bd37 gene present within the integrated expression construct (Figure 4B). In each of the experimental groups, the transgenic *T. theileri* was sustained in all animals throughout the 12 weeks after inoculation. This was without any adverse effects on animal health being detected. Moreover, the transgenic *T. theileri* could be recovered after several weeks of growth in cattle and was found to maintain Bd37 gene expression (Figure 5).

**Figure 6:**
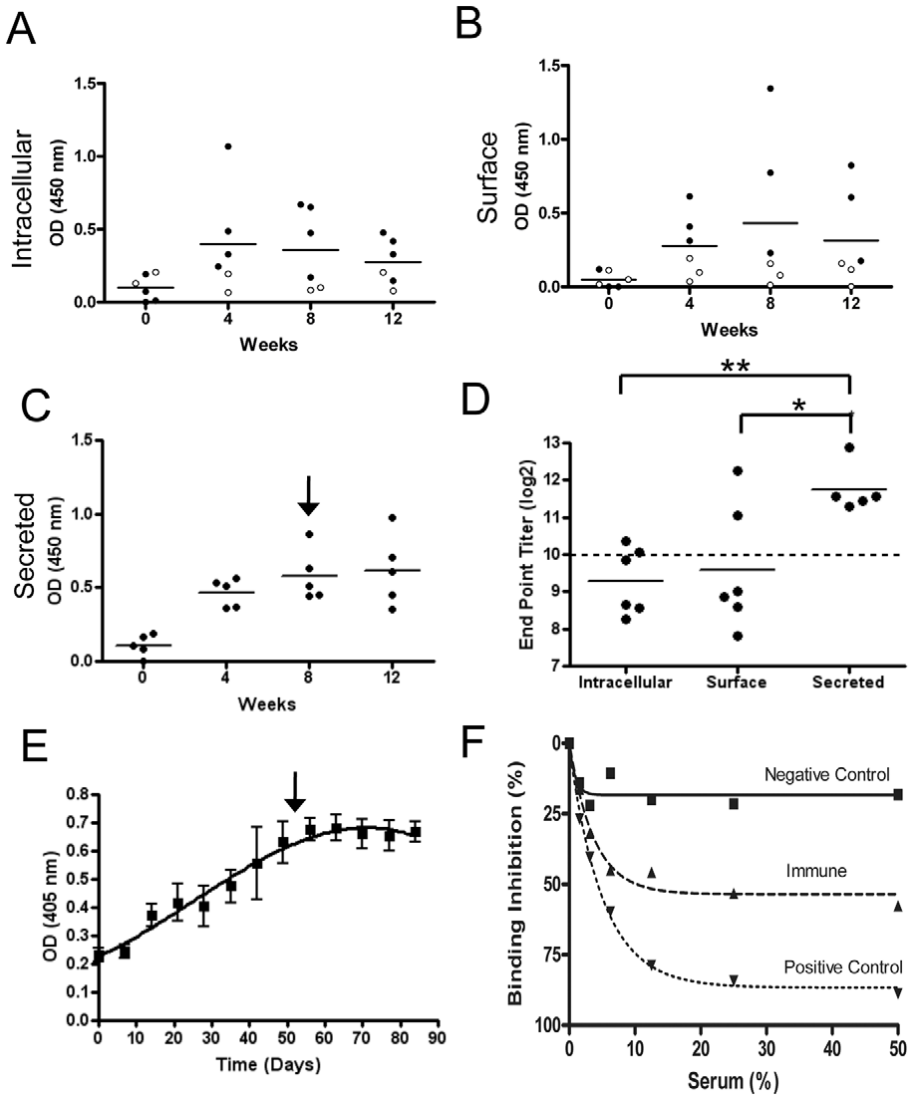
Inoculation with the vaccine vehicle stimulates an effective antibody response to the expressed antigen. (**A-C**) Quantification of anti-Bd37 antibody responses by ELISA assay demonstrate that calves exposed to Bd37 that is expressed at an internal (**A**) or surface location (**B**) on *T. theileri*, or is secreted (**C**), show sero-conversion (filled circles) within 12 weeks of inoculation. Animals that did not sero-convert are shown as open circles. (**D**) End-point titres measured for all experimental animals show that those cattle receiving secreted Bd37 achieved significantly higher antibody levels than the other treatment groups (* p<0.05, ** p<0.001). (**E**) Time-course of antibody response to secreted Bd37 vaccine vehicle in all cattle. (**F**) Sera from vaccinated animals are able to compete with an anti-Bd37 monoclonal antibody for ELISA binding; assay details are provided in the ‘Materials and Methods’. Vertical arrows in Panels C and E indicate inoculation of a further 1×10^6^ transgenic *T. theileri*.

To assess whether the inoculated animals were able to generate a specific immune response to the delivered antigen, serum samples were tested weekly for Bd37-specific IgG responses by ELISA. Under all treatment conditions, sero-conversion was observed, i.e. at least a 3-fold increase in ELISA OD compared to pre-immunisation control sera from the same animal (Figure 6A–C). Interestingly, the route of antigen expression affected both the sero-conversion frequency and the resulting antibody titre, with sero-conversion occurring in 4/6 animals receiving the cytosolically expressed antigen, 3/6 animals receiving the surface expressed antigen, and 5/5 animals receiving the secreted antigen. Moreover, the secreted antigen produced a significantly higher end-point serum titre than either the surface expressed antigen (Kruskal-Wallis one way ANOVA, p<0.05) or the cytosolically expressed antigen (p<0.001) (Figure 6D), with antibody levels continuing to increase for 60 days post inoculation before plateauing and remaining high for at least a further 24 days (Figure 6E). Inoculation of a further 1×10^6^ cells expressing the secreted antigen on week 8 had no detectable stimulatory effect on the levels of antibody generated (vertical arrow, Figure 6C, E).

To compare the antibody responses generated by transgenic *T. theileri* with conventional immunisation approaches, the antibody titres were assessed with respect to those established in a prime-boost vaccination study using the same antigen. This demonstrated that the levels of anti-Bd37 antibody stimulated by the transgenic *T. theileri* delivery system were equivalent to those generated by the same antigen delivered in conventional adjuvant, producing titres that are protective against *Babesia* in challenge trials (dashed line, Figure 6D). Furthermore, the immune response generated to the *T. theileri*-secreted Bd37 could effectively compete with a monoclonal antibody able to confer passive immunity to *Babesia* infection in a gerbil model (Figure 6F), demonstrating the recognition of common, potentially protective epitopes. Combined, these assays confirm that *T. theileri* represents an effective antigen delivery system able to generate sustained immune responses equivalent to, or exceeding, those generated by standard vaccination and at levels known to be protective against a target pathogen.

## Discussion

Our work describes a naturally non-pathogenic eukaryotic organism, *T. theileri*, engineered to deliver antigens and therapeutic proteins to cattle. Importantly, *T. theileri* is a flexible and adaptable system for the targeted expression of individual, or cocktails of multiple, heterologous proteins simultaneously. This allows for the targeting of different pathogens, or multiple defined antigens of a specific pathogen, providing an effective approach to limiting the transmission of disease from livestock to man, as well as maintaining bovine health and maximising bovine productivity. This system has many advantages over traditional vaccine delivery technologies, including the possibility of oral delivery, a natural route of *T. theileri* infection, and the potential for sustained immune stimulation through prolonged infection without adverse consequence on productivity or health. It also provides an explicit example of an unanticipated applied benefit derived from the extensive research investment in the basic biology of kinetoplastid pathogens.

A number of kinetoplastid parasites have been successfully transfected to drive ectopic expression of endogenous genes or heterologous expression of reporter genes[35] . In general, these have been used for the analysis of gene function in the transfected organisms or for protein expression in vitro. In particular, the kinetoplastid protozoa *T. brucei, Crithidia fasciculata, Leishmania amazonensis* and *Leishmania tarentolae* have each been engineered as eukaryotic vehicles for the expression of proteins which are appropriately post-translationally modified, that retain biological activity, or which are suitable for meaningful inhibition studies for drugs targeted against kinetoplastids [36]–[40]. In each case, the basic organisation of the expression vectors employed was similar. However, there is no predictable cross functionality of RNA processing signals . For example, in one study, transfection of a range of kinetoplastid protozoa with a common expression construct developed in *Leishmania major* resulted in reporter gene expression in other *Leishmania* spp., but not in *T. brucei* or *T. cruzi* [41].

The specificity of RNA processing signals among kinetoplastids necessitated that we isolated intergenic sequences from *T. theileri*. To our knowledge, no protein coding gene sequence have been characterised in these organisms to date. Despite this, we were able to make use of the tandem arrangement of several conserved housekeeping genes in trypanosomatids to isolate intergenic sequences between alpha and beta tubulin genes, beta and alpha tubulin genes, actin genes and the PFR genes. Conservation in these genes allowed successful amplification between genes using degenerate oligonucleotides. In each case, the predicted protein sequences were highly similar to those in other kinetoplastids over the sequenced region, this being reinforced by the reactivity of monoclonal antibodies specific for alpha tubulin, PFRA and BiP in *T. brucei* with *T. theileri* (Figure 1A, Figure 2 and data not shown). Extensive protein coding similarity is also evident from a whole genome analysis of *T. theileri* that we have recently completed (our unpublished observations). Despite this similarity in coding regions, there was no recognisable similarity in the intergenic regions of each gene between species. This emphasised the requirement to use endogenous intergenic sequences in the developed expression constructs. Indeed, transient transfection of *T. theileri* with a reporter construct generated for use in *T. brucei* did not result in detectable heterologous gene expression (unpublished observations). Cloning and sequencing of 5′RACE products derived from the actin gene transcript identified the SL sequence common to all trypanosomatid mRNAs. Interestingly, the sequence of the leader RNA did not match the SL sequence of a previous *T. theileri* isolate (K127), which was reported to exhibit a 1 nucleotide distinction from the sequence identified on the majority of trypanosomatid parasites . Instead, the leader sequence identified in the isolate of *T. theileri* used in our study matched exactly the trypanosomatid consensus and shared with *T. theileri* D30 . This suggests variation in this sequence amongst different *T. theileri* isolates.

Previous approaches have used kinetoplastid parasites as vehicles for protein expression *in vitro*, or for potential medicinal use via expression of biomolecules from attenuated pathogens *in vivo*. In these, and other cases of pathogen-based vaccine vectors (for example, using attenuated *Salmonella* , *Mycobacterium* , *E. coli* , *V. cholera*, *Listeria* or *Shigella*), the vehicles have a potential to revert to pathogenicity raising issues of long-term efficacy and safety. Moreover, recombination with non-attenuated pathogens can generate vaccine escape mutants, as observed after pneumococcal vaccination in the USA [42]. By exploiting an already non-pathogenic organism (*T. theileri*) for protein expression in its natural host, the possibility of reversion to, or unanticipated, pathogenicity is greatly reduced. Supporting this, the *T. theileri* isolate used in our studies has been maintained in tissue culture for over 6 years and yet, when inoculated into cattle, generated no ill effects and was sustained at low abundance throughout a 12-week trial. The transgenic parasites could also establish and be sustained in the context of a pre-existing natural infection. This is an important consideration since *T. theileri* is almost ubiquitous in cattle herds worldwide with, for example, 44%–80% incidence levels reported in New York State and up to 93% in Louisiana [43]. A survey of local adult cattle in Edinburgh revealed that all animals tested (16/16) were positive for *T. theileri* infection by the nested PCR assay for tubulin intergenic sequence (data not shown).

*T. theileri* has clear potential as a flexible vaccine vehicle, able to target a wide range of pathogens, including viruses (e.g. Foot and Mouth Disease Virus, Bovine Viral Diarrhea Virus), bacteria (e.g. *E. coli*, *Salmonella* spp., *Campylobacter* spp., *Brucella* spp. and mycobateria), ectoparasites (e.g. ticks, mites, lice) as well as pathogenic eukaryotic parasites (*Babesia* spp., *Neospora caninum, Coccidia* spp., *Trichomonas* spp. and helminths). However, *T. theileri* also has potential to deliver therapeutic proteins such as antimicrobials systemically in cattle, able to limit the impact of bacterial infections, for example. Furthermore, we propose that it may be possible to avoid the need to engineer transgenic cattle to express ApoLI or mutant variants [44], [45], allowing indigenous and disease-resistant African livestock to combat pathogenic trypanosome infection via *T. theileri* directed ApoL1 expression. Such an approach would require that the ApoL1 is expressed in a form that is non-immunogenic and active in cattle. Nonetheless, this could provide a simple and cost effective route to limiting the effects of trypanosome infection on the productivity or zoonotic potential of indigenous African cattle. This has the potential to increase their productivity and eliminate an important reservoir for human disease, alleviating poverty and disease in afflicted regions.

## Materials and Methods

### Ethics statement

Animal trials in this manuscript were reviewed and approved through the ethical review committee at Moredun Research Institute (Experiment approval no. E28/10). Experiments were carried out under a UK Home Office Licence (PPL 60/4044) in accordance with the UK Animals (Scientific Procedures) Act, 1986.

### Cell culture

Bovine conditioned media was produced by growing Madin-Darby Bovine Kidney (MDBK) cells in Eagle's Minimum Essential Medium with Earle's Balanced Salt Solution and sodium bicarbonate (Sigma, M2279) supplemented with 1% MEM non-essential amino acids (Invitrogen, 11140), 1% L-Glutamine solution (from 200 mM solution, Sigma, G7513), and 10% FCS until confluence (2–3 days). The MDBK-conditioned media was then harvested and filtered prior to use. *T. theileri* were cultured in 50% HMI-9 medium [46] supplemented with 20% FCS and 10% Serum^+^ and 50% MDBK-conditioned media as described above.

### Creation of recombinant cell lines

The plasmid backbone used for construction of all expression vectors was derived from the pGemT Easy plasmid (Promega). All elements of the vector were prepared through PCR of the noted sequence, followed by cloning into the vector backbone, and construction was accomplished through the insertion of appropriate restriction sites at the 5′ and 3′ ends of each segment. The segments used for the construction of the expression cassettes and the corresponding primers used in this study include: the 5′ fragment of the *T. theileri* SSU rRNA gene (SSU5-ApaI-For and SSU5-AvrII-Rev), the 3′ fragment of the *T. theileri* SSU rRNA gene (SSU3-PacI-For and 3SSU-Rev-XmaI), the trans-splicing addition site from the actin IR sequence (splice-AvrII-For and splice-FseI-Rev), the Bd37 coding sequence (Bd37-Core-F-FseI or Bd37-Core-F-AvrII and Bd37-Core-F-AvrII and Bd37-Core-R-HindIII), the CAT coding sequence (CAT For FseI or CAT For XhoI and CAT Rev XbaI or CAT Rev AscI), the blasticidin resistance cassette (BSDKpn-F and BSDBgl-R), the beta-alpha tubulin IR sequence (ba-tub-AscI-For or ba-tub-BglII and ba-tub-KpnI-Rev or ba-tub-PacI), the *T. brucei* BiP N-terminal fragment sequence (BiP-For-FseI or BiP-For-FseI-SpeI and BiP-Rev-XhoI), and the GPI addition signal (GPI-For-HindIII and GPI-Rev-AscI). In fusion protein cassettes, care was taken to preserve the reading frame and appropriate start and stop codons were provided. The plasmids containing the expression cassettes were then digested with appropriate restriction enzymes to liberate the plasmid backbone from the linear expression cassette, which was isolated via gel electrophoresis and gel purification. The linear cassette was then purified by ethanol precipitation and resuspended in 5 ml of TE buffer (1 mM Tris-HCl (pH 8) and 0.1 mM EDTA).

From a culture of logarithmically growing *T. theileri* parasites, 10 ml of culture at ∼5×10^5^ cells/ml were used for each transfection. Cells were centrifuged at 1000 × g, for 10 minutes at room temperature and resuspended in 1 ml of sterile PBS to wash the cells. Cells were then re-centrifuged and resuspended in 100 µl Ingenio transfection buffer (Mirus Bio). The cells were added to the prepared linear DNA and transferred to a cuvette for the Nucleofector II electroporation device (Lonza). Transfection was done with Nucleofector program X-001 (recommended for mouse CD8+ T cells) and cells transferred to a culture flask containing 10 ml of pre-warmed media and incubated for 24 hrs at 37°C, in 5 % CO_2_. Cells were then transferred to media containing the selective drug concentration (10 µg/ml of blasticidin) and plated using a variety of dilutions into a 24-well cell culture plate. After selection clones were recovered under normal cell culture conditions.

### Immunofluorescence assay

The localisation of the BiP-Bd37 fusion protein was determined using 2% paraformaldehyde fixed cells permeabilised, or not, with TBS: 0.1% Triton X-100 for 2 minutes. Cells were quenched for 30 minutes with TBS:0.1% glycine and then blocked for 1 hr with TBS: 1%BSA. Cells were then reacted with anti-BiP antibody (a gift of Jay Bangs, University of Wisconsin) diluted 1∶200 in TBS:1%BSA, washed three times with TBS and then incubated with anti-rabbit FITC conjugated antibody (Sigma) diluted 1∶100 inTBS:1%BSA. Prior to mounting in MOWIOL, cells were stained with 4′, 6-diamidino-2-phenylindole for 5 minutes to visualize cellular DNA.

### Experimental animals, sampling and DNA extraction

Calves (6-weeks old) were injected intravenously with 1×10^5^ *T. theileri* parasites. Blood samples were taken weekly into EDTA-containing tubes (Becton-Dickinson) and DNA extracted. DNA extraction from whole blood samples was performed as follows: 1 ml of blood was mixed thoroughly with 0.5 ml of RBC lysis buffer (0.32 M sucrose, 10 mM Tris-HCl pH 7.5, 5 mM MgCl_2_, 0.75% Triton X-100) in a microfuge tube. The samples were then centrifuged at 14, 000 rpm for 1 minute to pellet all cells and the supernatant removed. The pellets were repeatedly resuspended and recovered from 0.5 ml aliquots of RBC lysis buffer until no red blood cells were present. The resulting pellets were resuspended in 100 µl of lysis buffer (50 mM KCl, 10 mM Tris-HCl pH 8.3, 2.5 mM MgCl_2_, 0.1 mg/ml gelatin, 0.45% NP40, 0.45% Tween-20, 60 µg/ml proteinase K) and kept at 55°C for 60 minutes. The samples were then incubated at 95°C for 10 minutes prior to storage at −20°C until use.

### Nested PCR

Nested PCR reactions were designed to amplify either the *T. theileri* β-α tubulin intergenic sequence (to identify any *T. theileri* population) or the Bd37 coding sequence (specific for the vaccine vehicle). The primers used were as follows:

Tub Diagnostic F1: 5′-AGTAGCAACGACAGCAGCAGT-3′

Tub Diagnostic R1: 5′-GTAAAGTGTTTGAAGAAGAGCTCG-3′

Tub Diagnostic F2: 5′-CGATTCTCTTCGCCTGTTTGT-3′

Tub Diagnostic R2: 5′-ACTAACCGCGACCAAAGAAGT-3′

Bd37 Diagnostic F1: 5′-GCTCACAGGAGCCAGCAGCGG-3′

Bd37 Diagnostic R1: 5′-CCAGAGCTTTGAGATTAGCTGGTA-3′

Bd37 Diagnostic F2: 5′-ACGCAGCAAGGTGGTGCGAA-3′

Bd37 Diagnostic R2: 5′-AGCAAGGCCTCACCGCCCTTGGC-3′

Each 25 µl reaction contained the following components: 5 µl of template, 1X PCR buffer, 0.2 mM of each dNTP, 1.25 mM MgCl_2_, 0.4 µM of each primer and 0.25 U GoTaq Flexi DNA Polymerase (Promega). The first stage PCR reactions were heated to 95°C for 5 minutes, followed by 35 cycles of denaturation at 95°C for 30 seconds, annealing at 60°C for 45 seconds and elongation at 72°C for 45 seconds. Following the final cycle, the reactions were elongated for a further 4 minutes. The second stage nested PCR reaction was conducted using the same conditions with 5 µl of the first reaction as template. The resulting products were electrophoresed on a 1% Tris-acetic acid-EDTA agarose gel, stained with ethidium bromide and visualized.

### Serum preparation and ELISA assay

Serum samples were prepared from 10 ml of fresh blood and stored at −20°C until use. Recombinant (*E. coli*) expressed His-tagged Bd37 antigen was diluted to 5 µg per ml in coating buffer (0.01 M sodium carbonate pH 9.6), and 100 µl were added to each well of microtiter plates and incubated overnight at 37°C. The coating buffer was removed and 200 µl blocking buffer (10% horse serum in 10 mM PBS) were added, and incubated at 37°C for 60 minutes. The plates were washed 3 times with 200 µl washing buffer (10 mM PBS, pH 9.6 with 1% Tween-20). Bovine serum samples were diluted in blocking buffer as appropriate, and 100 µl were incubated in the coated and incubated at 37°C for 60 minutes. For standard ELISA plates were washed and incubated with HRP-conjugated anti-bovine IgG antibody diluted 1∶1000 in blocking buffer and incubated at 37°C for 60 minutes. For competitive ELISA assays, 100 µl of mouse monoclonal anti-Bd37 antibody (1 mg/ml diluted 1∶1000 in blocking buffer) were added to each well and incubated at 37°C for 60 minutes. Plates were washed and incubated with HRP-conjugated anti-mouse IgG antibody diluted 1∶1000 in blocking buffer and incubated at 37°C for 60 minutes. Plates were washed, treated with TMB supersensitive substrate (Sigma), stopped with 2M sulphuric acid and the OD was measured at 450 nm in an ELISA plate reader.

### Statistics

To analyse serum antibody titres, a Kruskal-Wallis one way ANOVA was used.

## Supporting Information

Text S1 **Supplementary information relating to the manuscript.**
(PDF)

## References

[1] Coker R, Rushton J, Mounier-Jack S, Karimuribo E, Lutumba P (2011). Towards a conceptual framework to support one-health research for policy on emerging zoonoses.. Lancet Infect Dis.

[2] Vanhamme L, Paturiaux-Hanocq F, Poelvoorde P, Nolan DP, Lins L (2003). Apolipoprotein L-I is the trypanosome lytic factor of human serum.. Nature.

[3] Xong HV, Vanhamme L, Chamekh M, Chimfwembe CE, Van Den Abbeele J (1998). A VSG expression site-associated gene confers resistance to human serum in *Trypanosoma rhodesiense*.. Cell.

[4] Kieft R, Capewell P, Turner CM, Veitch NJ, MacLeod A (2010). Mechanism of *Trypanosoma brucei gambiense* (group 1) resistance to human trypanosome lytic factor.. Proc Natl Acad Sci USA.

[5] Kennedy MJ (1988). Alberta. *Trypanosoma theileri* in cattle of central Alberta.. Can Vet J.

[6] Matthews DM, Kingston N, Maki L, Nelms G (1979). *Trypanosoma theileri* Laveran, 1902, in Wyoming cattle.. Am J Vet Res.

[7] Woo P, Soltys MA, Gillick AC (1970). Trypanosomes in cattle in southern Ontario.. Can J Comp Med.

[8] Villa A, Gutierrez C, Gracia E, Moreno B, Chacon G (2008). Presence of *Trypanosoma theileri* in Spanish Cattle.. Ann N Y Acad Sci.

[9] Niak A (1978). The incidence of *Trypanosoma theileri* among cattle in Iran.. Trop Anim Health Prod.

[10] Farrar RG, Klei TR (1990). Prevalence of *Trypanosoma theileri* in Louisiana cattle.. J Parasitol.

[11] Verloo D, Brandt J, Van Meirvenne N, Buscher P (2000). Comparative in vitro isolation of *Trypanosoma theileri* from cattle in Belgium.. Vet Parasitol.

[12] Ristic M, Trager W (1958). Cultivation at 37°C of a Trypanosome (*Trypanosoma theileri*) from Cows with Depressed Milk Production.. J Protozool.

[13] Doherty ML, Windle H, Voorheis HP, Larkin H, Casey M (1993). Clinical disease associated with *Trypanosoma theileri* infection in a calf in Ireland.. Vet Rec.

[14] Lanevschi-Pietersma A, Ogunremi O, Desrocher A (2004). Parasitemia in a neonatal bison calf.. Vet Clin Pathol.

[15] Seifi HA (1995). Clinical trypanosomosis due to *Trypanosoma theileri* in a cow in Iran.. Trop Anim Health Pro.

[16] Bose R, Friedhoff KT, Olbrich S, Buscher G, Domeyer I (1987). Transmission of *Trypanosoma theileri* to cattle by Tabanidae.. Parasitol Res.

[17] Robertsson JA, Lindberg AA, Hoiseth S, Stocker BA (1983). *Salmonella typhimurium* infection in calves: protection and survival of virulent challenge bacteria after immunization with live or inactivated vaccines.. Infect Immun.

[18] Hoiseth SK, Stocker BA (1981). Aromatic-dependent *Salmonella typhimurium* are non-virulent and effective as live vaccines.. Nature.

[19] Barletta RG, Snapper B, Cirillo JD, Connell ND, Kim DD (1990). Recombinant BCG as a candidate oral vaccine vector.. Res Microbiol.

[20] Chen TM, Mazaitis AJ, Maas WK (1985). Construction of a conjugative plasmid with potential use in vaccines against heat-labile enterotoxin.. Infect Immun.

[21] Radford KJ, Jackson AM, Wang JH, Vassaux G, Lemoine NR (2003). Recombinant *E. coli* efficiently delivers antigen and maturation signals to human dendritic cells: presentation of MART1 to CD8+ T cells.. Int J Cancer.

[22] Sizemore DR, Branstrom AA, Sadoff JC (1995). Attenuated *Shigella* as a DNA delivery vehicle for DNA-mediated immunization.. Science.

[23] Gull K (1999). The cytoskeleton of trypanosomatid parasites.. Annu Rev Microbiol.

[24] Borst P (1986). Discontinuous transcription and antigenic variation in trypanosomes.. Annu Rev Biochem.

[25] Tschudi C, Ullu E (1994). Trypanosomatid protozoa provide paradigms of eukaryotic biology.. Infect Agents Dis.

[26] Gibson W, Bingle L, Blendeman W, Brown J, Wood J (2000). Structure and sequence variation of the trypanosome spliced leader transcript.. Mol Biochem Parasitol.

[27] El-Sayed NM, Myler PJ, Blandin G, Berriman M, Crabtree J (2005). Comparative genomics of trypanosomatid parasitic protozoa.. Science.

[28] Bangs JD, Brouch EM, Ransom DM, Roggy JL (1996). A soluble secretory reporter system in *Trypanosoma brucei*. Studies on endoplasmic reticulum targeting.. J Biol Chem.

[29] Delbecq S, Precigout E, Vallet A, Carcy B, Schetters TP (2002). *Babesia divergens*: cloning and biochemical characterization of Bd37.. Parasitol.

[30] Brown WC, Norimine J, Goff WL, Suarez CE, McElwain TF (2006). Prospects for recombinant vaccines against *Babesia bovis* and related parasites.. Parasite Immunol.

[31] Goff WL, Wagner GG, Craig TM (1984). Increased activity of bovine ADCC effector cells during acute Babesia bovis infection.. Vet Parasitol.

[32] Goff WL, Wagner GG, Craig TM, Long RF (1984). The role of specific immunoglobulins in antibody-dependent cell-mediated cytotoxicity assays during Babesia bovis infection.. Vet Parasitol.

[33] Precigout E, Delbecq S, Vallet A, Carcy B, Camillieri S (2004). Association between sequence polymorphism in an epitope of Babesia divergens Bd37 exoantigen and protection induced by passive transfer.. Int J Parasitol.

[34] Hadj-Kaddour K, Carcy B, Vallet A, Randazzo S, Delbecq S (2007). Recombinant protein Bd37 protected gerbils against heterologous challenges with isolates of *Babesia divergens* polymorphic for the bd37 gene.. Parasitol.

[35] Clayton CE (1999). Genetic manipulation of kinetoplastida.. Parasitol Today.

[36] Biebinger S, Wirtz LE, Lorenz P, Clayton C (1997). Vectors for inducible expression of toxic gene products in bloodstream and procyclic *Trypanosoma brucei.*. Mol Biochem Parasitol.

[37] Kelly JM, Ward HM, Miles MA, Kendall G (1992). A shuttle vector which facilitates the expression of transfected genes in *Trypanosoma cruzi* and *Leishmania*.. Nucl Acids Res.

[38] Tetaud E, Lecuix I, Sheldrake T, Baltz T, Fairlamb AH (2002). A new expression vector for *Crithidia fasciculata* and *Leishmania*.. Mol Biochem Parasitol.

[39] Coburn CM, Otteman KM, McNeely T, Turco SJ, Beverley SM (1991). Stable DNA transfection of a wide range of trypanosomatids.. Mol Biochem Parasitol.

[40] Furger A, Jungi TW, Salomone JY, Weynants V, Roditi I (2001). Stable expression of biologically active recombinant bovine interleukin-4 in *Trypanosoma brucei*.. FEBS letters.

[41] Clayton CE, Ha S, Rusche L, Hartmann C, Beverley SM (2000). Tests of heterologous promoters and intergenic regions in *Leishmania major*.. Mol Biochem Parasitol.

[42] Brueggemann AB, Pai R, Crook DW, Beall B (2007). Vaccine escape recombinants emerge after pneumococcal vaccination in the United States.. PLoS Pathog.

[43] Schlafer DH (1979). *Trypanosoma theileri*: a literature review and report of incidence in New York cattle.. Cornell Vet.

[44] Thomson R, Molina-Portela P, Mott H, Carrington M, Raper J (2009). Hydrodynamic gene delivery of baboon trypanosome lytic factor eliminates both animal and human-infective African trypanosomes.. Proc Natl Acad Sci U S A.

[45] Lecordier L, Vanhollebeke B, Poelvoorde P, Tebabi P, Paturiaux-Hanocq F (2009). C-terminal mutants of apolipoprotein L-I efficiently kill both *Trypanosoma brucei brucei* and *Trypanosoma brucei rhodesiense*.. PLoS Pathog.

[46] Hirumi H, Hirumi K (1989). Continuous cultivation of *Trypanosoma brucei* blood stream forms in a medium containing a low concentration of serum protein without feeder cell layers.. J Parasitol.

