# Supplementary material for: Targeting Cattle-Borne Zoonoses and Cattle Pathogens Using a Novel Trypanosomatid-Based Delivery System
Source: PLoS Pathog. 2011 Oct 27;7(10):e1002340. doi: 10.1371/journal.ppat.1002340 (PMC3203185; doi:10.1371/journal.ppat.1002340)
Supplement: Text S1 — Supplementary information relating to the manuscript. (PDF) [file ppat.1002340.s001.pdf]

TEXT S1: Supplementary Data

Figure S1: The morphology of *Trypanosoma theileri* in culture at different cell densities

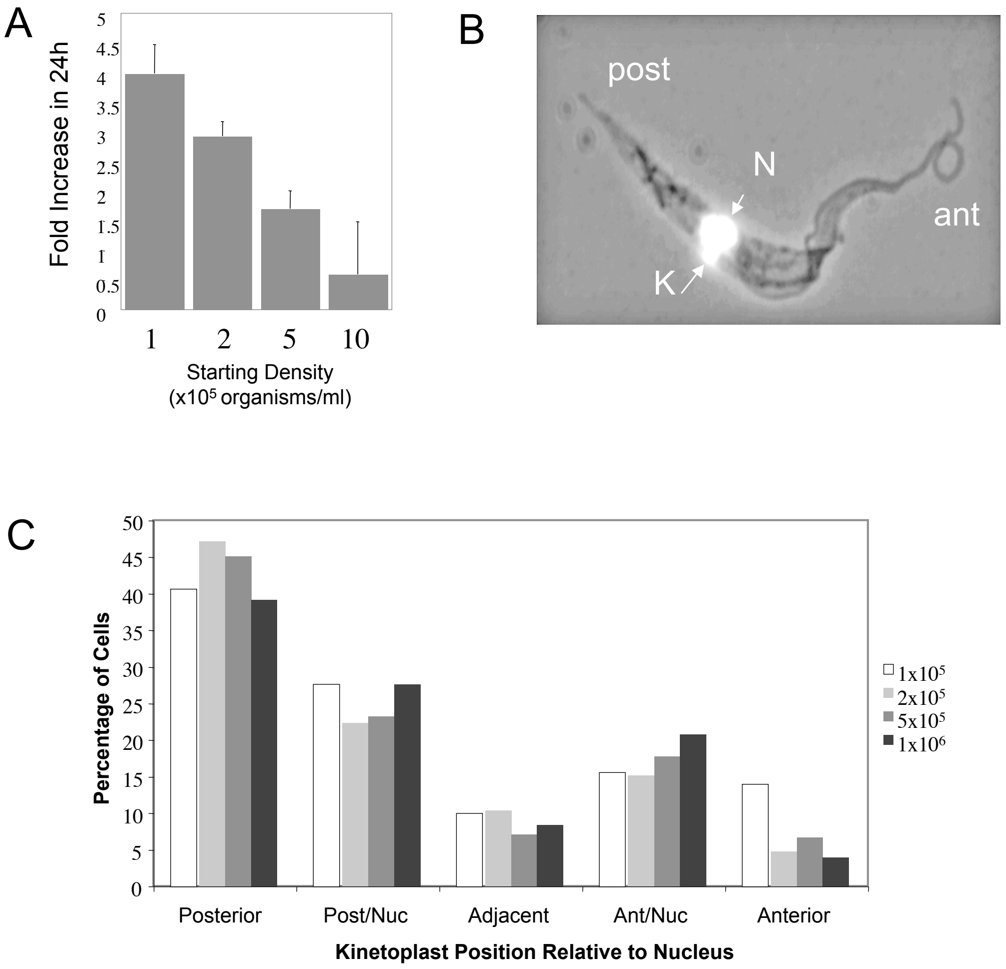

A. Growth of *T. theileri* when sub-passaged at differing initial densities ($1x10^5$/ml; 2 $x10^5$/ml; $5x10^5$/ml and $1x10^6$/ml). Parasites were diluted every 24 h back to the initial density and the rate of growth plotted. Optimal proliferation was observed when parasites were passaged daily to $1x10^5$/ml.

B. A phase contrast image of a bloodstream form of *T. theileri* grown in long term culture. The position of the mitochondrial genome (Kinetoplast; K) is indicated, in this case overlying the nucleus (N). In other cells the kinetoplast was observed to lie toward the posterior or anterior of the cell.

C. Relative proportion of different morphological forms of *T. theileri* in culture in relation to the passage density of the cell population. Posterior: the kinetoplast was positioned between the nucleus and posterior of the cell but did not lie over the nucleus; Post-nuc, the kinetoplast was positioned over the nucleus, but displaced toward the posterior; Nucleus: the kinetoplast was positioned exactly over the centre of the nucleus; Ant-nuc: the kinetoplast was positioned over the nucleus but displaced toward the anterior; Anterior: the kinetoplast was positioned toward the anterior of the cell and did not overlie the nucleus.

Figure S2 : The derived spliced leader sequence of the *T. theileri* isolate used in these studies in comparison to other trypanosomatids

Comparison of longest splice leader obtained by 5’-RACE with those of Gibson *et al.* (2000)

Trypanosoma consensus SL	AACTAACGCTATTATTGATACAGTTTCTGTACTATATTG
T. theileri K127 SL	AACTAACGATATTATTGATACAGTTTCTGTACTATATTG
T. theileri D30 SL	AACTAACGCTATTATTGATACAGTTTCTGTACTATATTG
T. theileri (this study)	------CGCTATTATTGATACAGTTTCTGTACTATATTG
T. brucei SL	AACTAACGCTATTATTAGAACAGTTTCTGTACTATATTG

Sequence of the partial SL sequence from *T. theileri* in this study in comparison to SL sequences from two previously isolated *T.theileri* lines (K127, D3), and *Trypanosoma brucei*. The sequence was derived by 5’RACE analysis of the *T. theileri* actin transcript.

Figure S3: The intergenic sequence between the *T. theileri* beta and alpha tubulin genes aligned with the equivalent intergenic sequence from *T. brucei*

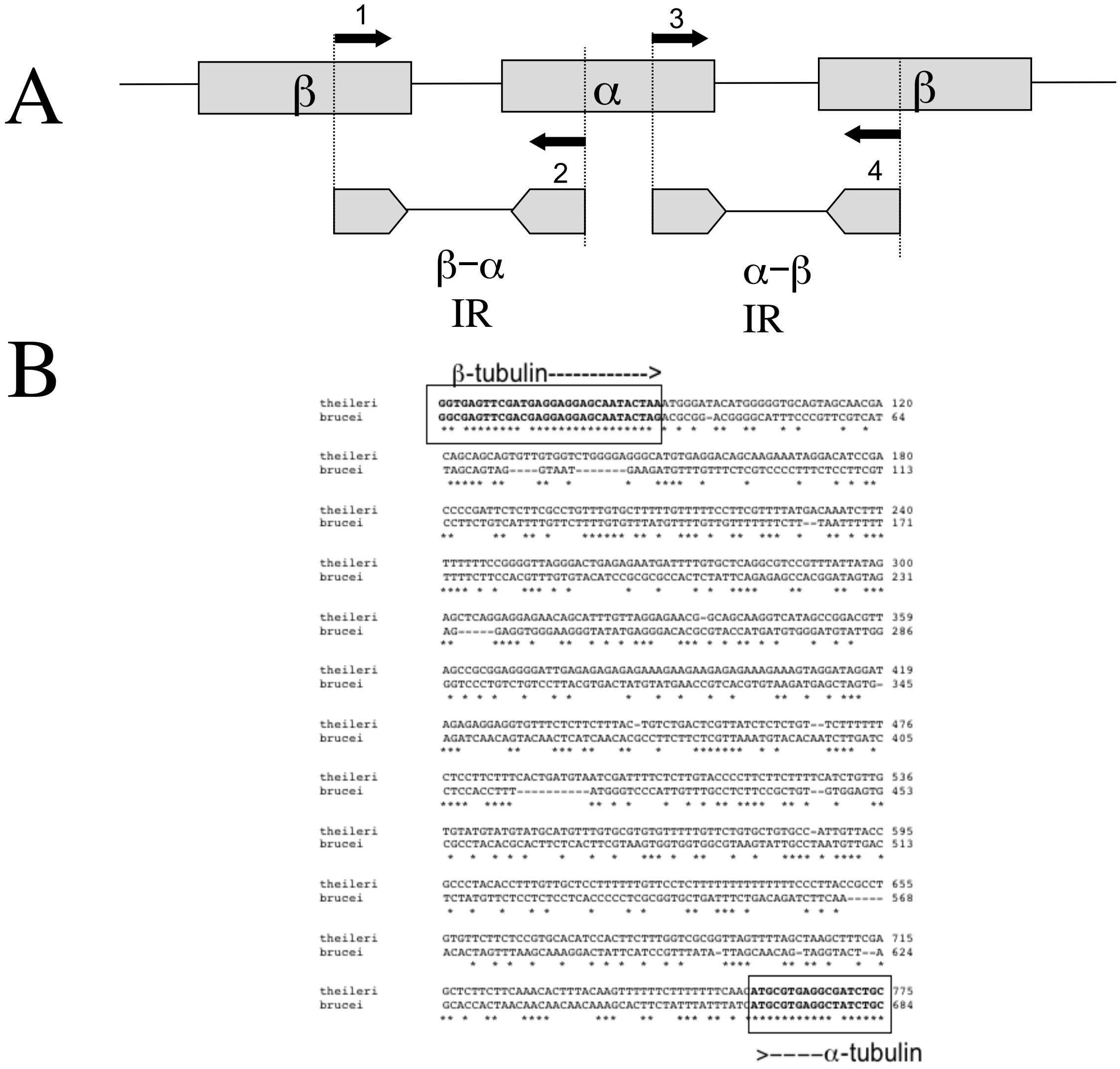

A. Amplification of the intergenic region (IR) separating the *T. theileri* beta and alpha tubulin, or alpha and beta tubulin genes. In each case, degenerate oligonucleotide primers based on the conserved protein-coding region of alpha or beta tubulin from different kinetoplastid parasites were used. Primer 1, β-tubulin F; primer 2, α-tubulin R; primer 3, α-tubulin F, primer 4, β-tubulin R.
B. Sequence comparison of the β tubulin and α tubulin intergenic regions of *T. theileri* and *T. brucei*. The 3' end of the coding sequence of β tubulin is boxed, as is the 5' end of the α tubulin coding sequence.

Figure S4: The polyadenylation site within the beta tubulin intergenic sequence used for the various expression constructs described in the manuscript.

Mapping of the polyadenylation sites used for each expression construct. Polyadenylation sites were mapped as detailed in Mayho M, Fenn K, Craddy P, Crosthwaite S, Matthews K. Nucleic Acids Res. 2006; 34(18):5312-24. Specifically, RT-PCR on RNA from each cell line was carried out. The 3'end of the *BD37* transcript was amplified using a gene-specific oligonucleotide hybridising to the Bd37 gene coding region (5' AGGTCAGGGATCTAGTCCTAAG 3') and a 3' oligo-dT ADAPT oligonucleotide hybridising to the poly(A) tail (5' GGC CAC GCG TCG ACT AGT ACT TTT TTT TTT TTT TT 3'). Amplified products were then subjected to a second round of nested amplification using an oligonucleotide hybridising to the *Bd37* coding region (5' TGCCATTGTCTTCGGTATCATTG 3') and the primer AUAP (5' GGC CAC GCG TCG ACT AGT AC 3'), which binds to the specific oligonucleotide sequence incorporated into the 5' end of the ADAPT primer [31]. The resulting products were gel purified and sequenced to determine the site of polyadenylation. Mapping of the *T. theileri* beta tubulin intergenic region was carried out identically using beta tubulin gene specific primers (Round 1; 5' GTCCAACATGAACGATCTCGTG-3'; Round 2; 5' GGGTGAGTTCGATGAGGAGGAG-3').

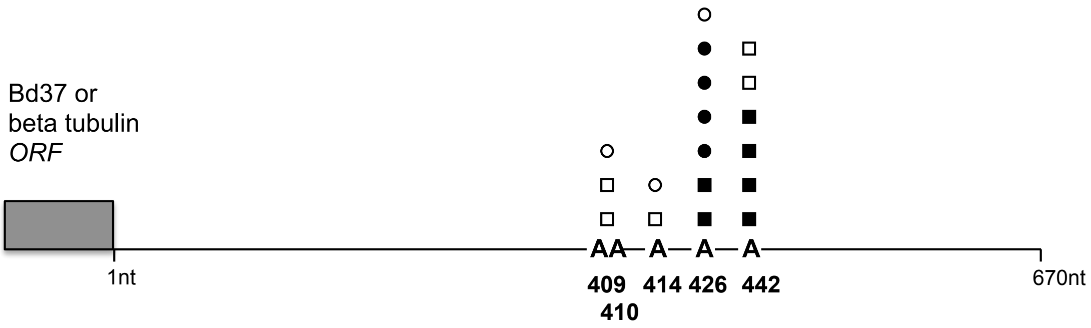

Beta-Alpha Tubulin Intergenic Region

Internally expressed Bd37 mRNA : Filled squares
Secreted Bd37 mRNA: Empty squares
Surface expressed Bd37 mRNA: Filled circles
Beta tubulin mRNA: empty circles
